# Examining the effect of a wearable, anxiety detection technology on improving the awareness of anxiety signs in autism spectrum disorder: a pilot randomized controlled trial

**DOI:** 10.1186/s13229-021-00477-z

**Published:** 2021-11-14

**Authors:** Jenny Nguyen, Robyn E. Cardy, Evdokia Anagnostou, Jessica Brian, Azadeh Kushki

**Affiliations:** 1 Bloorview Research Institute, Holland Bloorview Kids Rehabilitation Hospital, 150 Kilgour Rd, Toronto, ON M4G 1R8 Canada; 2 Institute of Biomedical Engineering, University of Toronto, 64 College St, Toronto, ON Canada; 3 Department of Paediatrics, University of Toronto, 555 University Avenue, Toronto, ON Canada

**Keywords:** Autism, ASD, Anxiety, Wearables, Intervention

## Abstract

**Background:**

Anxiety is prevalent in autism spectrum disorder (ASD) and can negatively impact physical and mental health. Self-awareness of anxiety signs is a key barrier to success of anxiety interventions for many children.

**Methods:**

To address this, we conducted a randomized controlled trial to assess whether the Anxiety Meter, a wearable, real-time anxiety detection technology, can improve awareness of anxiety symptoms and the initiation of relaxation techniques in children with ASD. Twenty-eight children with ASD were trained on the use of the Anxiety Meter and taught a diaphragmatic breathing relaxation technique over three visits. On the fourth visit, participants were randomized to either receive feedback of their anxiety level or no feedback from the Anxiety Meter while completing a stress-eliciting task (public speaking) and asked to engage in deep breathing if anxious.

**Results:**

Feedback from the Anxiety Meter was associated with increased likelihood of initiating deep breathing in response to anxiety.

**Limitations:**

Limitations include the small sample size, imbalanced group matching for IQ and sex, and the controlled-laboratory settings which limit the statistical power and generalizability of the results to real-world settings.

**Conclusions:**

Although these results are limited by the relatively small sample size, they support the feasibility of using a wearable device and real-time feedback to improve anxiety symptom awareness.

*Trial Registration* ClinicalTrials.gov Identifier: NCT02160691, registration date: 06/05/2014.

## Introduction

Anxiety is a prevalent, persistent, and disabling comorbidity associated with autism spectrum disorder (ASD). Anxiety concerns in ASD persist over the life span [1] and occur across all levels of functioning [2]. Anxiety profoundly impacts the psychosocial development and physical and mental health of children and interacts with the core domains of ASD to increase functional impairment [3].

Treatment of anxiety in ASD remains a significant clinical challenge. There is evidence to suggest that cognitive-behavioural therapy (CBT) may be effective for a subgroup of children with ASD with higher verbal ability; however, the optimal response to CBT is contingent upon children’s capacity for introspection, self-identification of emotional states, and communication—domains that are commonly impaired in ASD [4–6]. ASD is also associated with difficulty in self-awareness of physiological states [7], an additional barrier to mastering physiological awareness and management components of CBT. To address this gap, we evaluated the efficacy of a wearable technology, the Anxiety Meter, in improving awareness of anxiety signs in children with ASD. The Anxiety Meter uses wearable sensors to sense changes in heart rate and translate these into a visual display of anxiety level which is presented to the user on a tablet.

## Methods

### Trial design

The study was a parallel randomized controlled trial consisting of three training and one intervention visit to our laboratory at Holland Bloorview Kids Rehabilitation Hospital. Participants were informed during consent that they would be randomized in a 1:1 fashion to either receive feedback on their anxiety level (treatment group) or no feedback (control group) from Anxiety Meter during the intervention visit. Due to the nature of the intervention, participants and investigators were not blind to the treatment allocation.

The study protocol was approved by Holland Bloorview’s research ethics board. Participants and their caregivers provided informed consent and assent as appropriate.

### Participants

A total of 38 participants were recruited through the Province of Ontario Neurodevelopmental Disorders (POND) Network. Inclusion criteria were age between 8 and 18 years, primary diagnosis of ASD supported by the Autism Diagnostic Observation Schedule (ADOS-2) [8] and the Autism Diagnostic Interview-revised (ADI-R) [9], full-scale IQ greater than 50, and ability to complete modules three or four of the ADOS-2. Our exclusion criteria were taking beta-blockers (which can affect heart rate), previous experience with CBT, or starting new treatments within four weeks of study enrolment.

### The anxiety meter

The version of the Anxiety Meter used in the study employed a wearable Shimmer2 unit (Shimmer Sensing Ltd.) connected via Bluetooth to record electrocardiogram (ECG) and respiration signals at a frequency of 256 Hz [10]. Electrodes were placed according to the Shimmer ECG User Guide with a bipolar limb leads system [11]. The ECG data were bandpass-filtered between 5 and 15 Hz and processed to remove noise and extract heart rate [12] and used to compute real-time anxiety level based on an established algorithm [13]. Anxiety level was displayed on a vertical colour gradient scale (green: calm, yellow: rising anxiety level, and red: anxious) on a tablet screen in the participant’s field of view. A horizontal bar moved up and down along the colour gradient to indicate current level of arousal. A “blob” character presented over the horizontal bar, sound notification, and vibration signals alerted the participants each time their anxiety level reached the yellow zone or above.

### Training

During the first three visits, all participants received Anxiety Meter training (based on a training script) and were taught diaphragmatic breathing by a psychologist or psychology intern trained in the Facing Your Fears program [14]. During the first visit, participants also watched a 15-min baseline video and completed an anxiety-eliciting task (Stroop Color-Word Interference task [15]). Participants were excluded from further participation in the study if they did not meet eligibility criteria for physiological arousal in response to the anxiety-eliciting task (minimum of two beats per minute increase in average heart rate during the Stroop task compared to baseline). At the end of the third visit, the psychologist or psychology intern determined if participants mastered the breathing technique and use of the Anxiety Meter based on clinical judgement. If the relaxation technique was not mastered by visit three, the participant was fully excluded from further participation in the study and was not scheduled for a fourth visit.

### Intervention

**Fig. 1:**
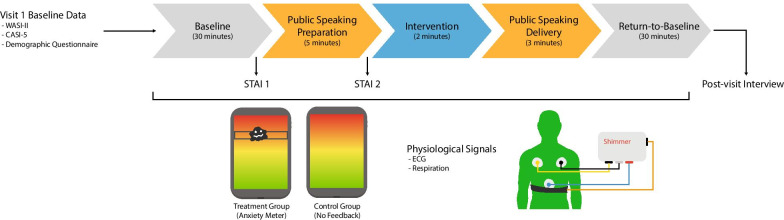
Schematic outline of protocol for visit 4. WASI-II: Wechsler Abbreviated Scale of Intelligence, Second Edition; CASI-5: Child and Adolescent Symptom Inventory-5; STAI: State-Trait Anxiety Inventory; ECG: electrocardiogram

At the outset of the intervention visit (visit four; Fig. 1), all participants were reminded that they can use deep breathing techniques throughout the study visit. Participants then watched a 30-min baseline video, after which participants were given five minutes to prepare a talk for a public speaking task [16]. Following preparation, participants completed a two-minute intervention period where they were instructed to reflect on their anxiety level. Both the treatment and control groups had an Anxiety Meter tablet on a stand in front of them during the intervention period. However, only participants allocated to the treatment group received feedback from the Anxiety Meter during this period. The Anxiety Meter provided to the control group did not provide real-time arousal feedback (horizontal bar displaying level of arousal was in a fixed position in the green (calm) zone throughout). Participants then delivered a three-minute speech to three strangers. Finally, participants watched a fifteen-minute return-to-baseline video.

### Measures

The primary outcome was the proportion of participants initiating diaphragmatic breathing during the intervention period. This was measured through self-report, supported by changes in respiration amplitude and rate. As exploratory outcome measures, we examined cardiac response (average heart rate) and self-reported anxiety symptoms assessed by change in the State-Trait Anxiety Inventory (STAI [17]) score from baseline to the public speaking preparation task, and by participants responses to a brief subjective experience interview at the end of visit four). See Table 1 for the interview question prompts.

**Table 1:** Post-visit four interview questions

	Interview question
1	Did you use your deep breathing when you started to feel anxious?
2	Why or why not?
3	If you used your deep breathing, what helped you remember to use it?
4	Did using it help you feel calmer?

IQ was measured using the Wechsler Abbreviated Scale of Intelligence, Second Edition [18]. General and separation anxiety, as well as attention-deficit symptom severity, were measured using the Child and Adolescent Symptom Inventory-5 (CASI-5 [19]).

Raw ECG signals were cleaned, and R-peaks were detected using the approach described by Pan and Tompkins [12]. Heart rate, measured in beats per minute, was calculated based on the R-R intervals.

A fifth order 2 Hz low-pass Butterworth filter was used to clean and preprocess respiration signals. False positive outliers (i.e. extrema in the respiration signal that had a smaller amplitude than 30% of the average amplitude) were removed [20]. Mean respiration rate reported in breaths per minute and mean respiration amplitude reported in log(millimetres) were calculated.

Anxiety meter level was also included as a physiological signal. Scores range from 0 (indicating calm) to 100 (indicating anxious).

### Analyses

#### Proportion of responders

Fisher's exact test was used to compare the number of responders (i.e. those who initiated the breathing technique) in the treatment and control groups. Logistic regression was used to quantify the effect of group on initiation of deep breathing while controlling for age, sex, and full-scale IQ.

#### Physiological signals

To confirm that the anxiety-eliciting (i.e. speech preparation and speech delivery) did elicit a state of arousal in participants, we conducted repeated measures linear mixed effects. The mixed effects analysis was used to examine the effect of group (i.e. anxiety meter group vs. control) and event (i.e. Baseline one and Intervention) on physiological measures (i.e. anxiety meter level, heart rate, high-frequency heart rate variability, respiration amplitude and respiration rate). Age, sex, and full-scale IQ were included as covariates for the linear models. A separate model was fitted for each of the signals measured (i.e. Anxiety Meter level, heart rate, respiration amplitude and respiration rate). Due to non-normality, respiration amplitude was transformed logarithmically.

## Results

**Fig. 2:**
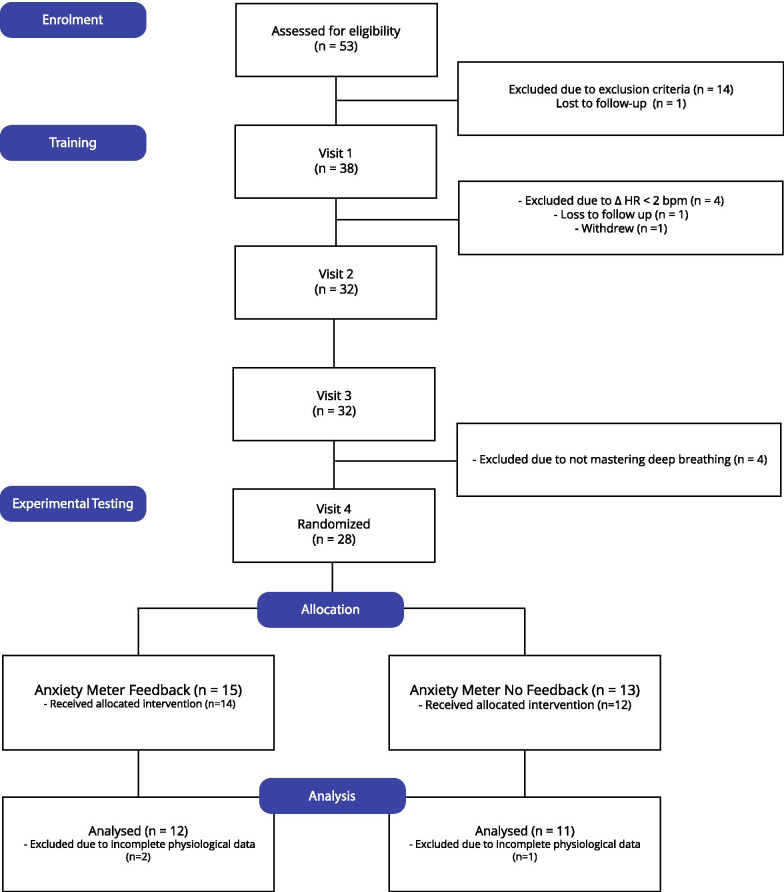
Consolidated Standards of Reporting Trials (CONSORT) flow diagram from enrolment to analysis. HR: heart rate; bpm: beats per minute; Δ: change

Fifty-three children were assessed for eligibility, and 38 met criteria and were enrolled in the study. After the first visit, four participants were screened out as they did not meet eligibility criteria for physiological arousal in response to the anxiety-inducing task (minimum of two beats per minute increase in average heart rate during the Stroop task compared to baseline). One participant was lost to follow-up and another participant withdrew. After the third visit, four participants were screened out because they did not master the breathing technique. Thus, 28 participants were eligible for the fourth visit. At the intervention session, two participants (one treatment and one control) did not complete the public speaking delivery task due to excessive anxiety. Thus, a total of 26 participants received the allocated intervention (Table 2). Two participants in the treatment group and one control participant were excluded from the secondary analysis due to technical difficulties (Fig. 2).

**Table 2:** Participant characteristics

	Treatment (n = 14)	Control (n = 12)
Age	14.2 ± 1.9	13.0 ± 3.2
Sex (male/female)	9:5	7:5
Full-scale IQ	107.0 ± 14.4	89.2 ± 18.6
CASI-5: generalized anxiety	72.0 ± 13.9	65.4 ± 14.9
CASI-5: separation anxiety	55.0 ± 17.5	59.8 ± 18.4
CASI-5: attention and hyperactivity	71.1 ± 8.8	63.9 ± 11.5
Responders (yes/no)	14:0	4:8

The statistics are reported as mean and standard deviation

### Proportion of responders

The proportion of participants who initiated deep breathing was significantly higher in the treatment group compared to the controls (responders/non-responders for Anxiety Meter group = 14:0, control group = 4:8; Fisher’s exact, *p* < 0.001). Of the eight non-responders (did not initiate deep breathing), none of them reported feeling calmer. Of those who did initiate deep breathing, 11/14 and 4/4 reported feeling calmer after deep breathing, in the Anxiety Meter and control groups, respectively. In the Anxiety Meter group, 11/14 participants reported that the Anxiety Meter reminded them to initiate deep breathing.

To account for imbalances in sex between the control and Anxiety Meter groups, we conducted separate analyses in male and female groups. The proportion of participants who initiated deep breathing in the treatment group compared to the control group was only higher for the male subgroup (male: Anxiety Meter: 9:0, control 5:2, Fisher’s exact, *p* = 0.005; female: Anxiety Meter: 5:0, control 2:3, Fisher’s exact, *p* = 0.167).

The results of logistic regression indicated that the logistic model explained 53.2% of the variance (*F*(4, 21) = 5.97, *p* = 0.0023). The Anxiety Meter group was more likely to initiate deep breathing (*β* = 0.693, OR = 2.00, 95% Confidence Interval (CI) = [0.340, 1.046], *p* = 0.001). Age, male gender, and full-scale IQ were not significant in the model.

#### Initiation of deep breathing in responders versus non-responders

Comparison of the respiration data between the intervention and anxiety task conditions was consistent with the initiation of deep breathing in the responder group (respiration amplitude: 18.6% increase in responders, 2.4% decrease in non-responders; respiration rate: 0.6% decrease in responders, 9.0% increase in non-responders). However, the difference in respiration amplitude and respiration rate between responders and non-responders was non-significant (*t* = 1.18, *p* = 0.640, 95% CI = [− 16.17, 58.27], *dof* = 21, and *t* =  − 0.47, *p* = 0.252, 95% CI = [− 51.77, 32.54], *dof* = 21, respectively).

### Effect of the anxiety paradigm

**Fig. 3:**
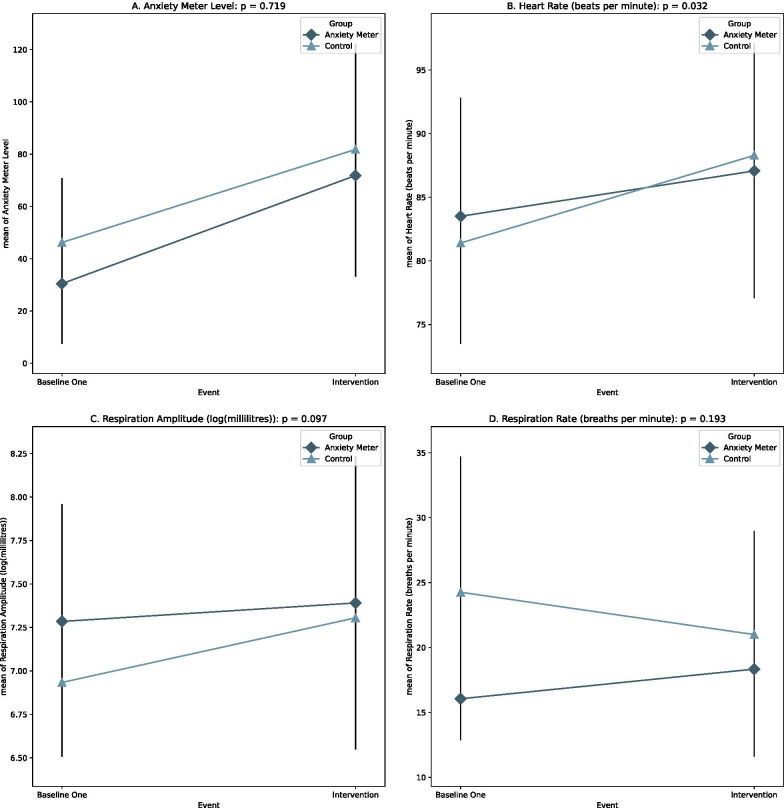
Group–event interaction effect for all signals measured. Error bars = standard deviation

Confirming that the study’s tasks did elicit states of arousal in participants, there was a significant increase in Anxiety Meter-indicated levels and all physiological measures, except respiration rate, from baseline to the intervention period (anxiety meter level: *β* = 38.61, 95% CI = [22.9, 54.33], *p* < 0.0001, heart rate: *β* = 5.16, 95% CI = [3.52, 6.80], *p* < 0.0001, respiration amplitude: *β* = 0.23, 95% CI = [0.07, 0.40], *p* = 0.005). The effect of group was not significant (Fig. 3).

Regarding covariates, age and full-scale IQ did not significantly predict anxiety level, heart rate, or respiration amplitude. Male sex (*β* = 0.67, 95% CI = [0.15, 1.20], *p* = 0.012) was associated only with elevated respiration amplitude.

## Discussion

In this study, we evaluated the efficacy of a wearable technology, the Anxiety Meter, to improve awareness of anxiety states in children with ASD. Our results showed that using the Anxiety Meter was associated with an increased likelihood of initiating calming strategies under laboratory-induced stress conditions. Our results also provide some evidence that the Anxiety Meter modulates the change in heart rate for anxiety-eliciting tasks. From baseline one to the intervention period, participants from both groups experienced an increase in heart rate, which is a physiological marker of anxiety [21]. The extent of heart rate increase was modulated by the group. The treatment group experienced a reduced increase in heart rate when compared to the control group. These results, along with self-reports of increased calmness, suggest that awareness of anxiety state in conjunction with initiation of deep breathing may help reduce anxiety. However, we did not find a significant group–event interaction effect for respiration amplitude and rate.

This suggests that physiologically-informed alerts may be a promising approach for improving awareness of anxiety signs. This is particularly encouraging for children with ASD for whom difficulties with self-awareness is a barrier to benefiting from existing anxiety interventions. Another benefit of this approach is that physiological alerts can be delivered in real-time and in situ through inexpensive and commercially-available devices such as smartwatches. This can enable the integration of anxiety interventions in everyday situations, and continuous reinforcement of use of therapeutic techniques across multiple settings. In our study, the Anxiety Meter alerts were provided to the children directly. It is also possible to provide these to caregivers so that de-escalation strategies can be initiated in a timely manner.

## Limitations

The findings of this study must be interpreted in the context of several limitations. First, our sample size was relatively small, and our statistical analyses were underpowered. Second, despite the randomized assignment of participants, the treatment and control groups were imbalanced in terms of IQ and sex, although the conclusions remained consistent when the groups were matched. Third, responses to public speaking tasks similar to the Trier Social Stress Test [22] may be altered in ASD due to reduced social evaluative threat [23]. Fourth, given the small sample size, the noise within the physiological signals, and the short intervention period, it was difficult to discern a difference between the treatment and control groups. Future studies investigating the impact of the Anxiety Meter on physiological signals collected over a longer intervention period in larger samples are necessary. Lastly, the controlled-laboratory settings limit the generalizability of the results to real-world settings. Future studies will explore the real-world use and feasibility of using the Anxiety Meter in clinical and educational settings.

## Conclusions

The results of this study support the preliminary efficacy of using the Anxiety Meter to improve awareness of anxiety signs in children with ASD. Future studies are needed to replicate these results in larger samples and real-world environments.

## Data Availability

The datasets used and/or analysed during the current study are available from the corresponding author on reasonable request.

## Abbreviations

ADI-R: Autism diagnostic interview-revised
ADOS-2: Autism Diagnostic Observation Schedule, Second Edition
ASD: Autism spectrum disorder
BPM: Beats per minute
CASI-5: Child and Adolescent Symptom Inventory-5
CBT: Cognitive-behavioural therapy
ECG: Electrocardiogram
STAI: State-Trait Anxiety Inventory
WASI-II: Wechsler Abbreviated Scale of Intelligence, Second Edition
